# The AgI/II Family Adhesin AspA Is Required for Respiratory Infection by *Streptococcus pyogenes*

**DOI:** 10.1371/journal.pone.0062433

**Published:** 2013-04-30

**Authors:** Linda Franklin, Angela H. Nobbs, Laura Bricio-Moreno, Christopher J. Wright, Sarah E. Maddocks, Jaspreet Singh Sahota, Joe Ralph, Matthew O’Connor, Howard F. Jenkinson, Aras Kadioglu

**Affiliations:** 1 Department of Infection, Immunity and Inflammation, University of Leicester, Leicester, United Kingdom; 2 School of Oral and Dental Sciences, University of Bristol, Bristol, United Kingdom; 3 Department of Clinical Infection, Microbiology and Immunology, Institute of Infection and Global Health, University of Liverpool, Liverpool, United Kingdom; Wake Forest University School of Medicine, United States of America

## Abstract

*Streptococcus pyogenes* (GAS) is a human pathogen that causes pharyngitis and invasive diseases such as toxic shock syndrome and sepsis. The upper respiratory tract is the primary reservoir from which GAS can infect new hosts and cause disease. The factors involved in colonisation are incompletely known however. Previous evidence in oral streptococci has shown that the AgI/II family proteins are involved. We hypothesized that the AspA member of this family might be involved in GAS colonization. We describe a novel mouse model of GAS colonization of the nasopharynx and lower respiratory tract to elucidate these interactions. We used two clinical M serotypes expressing AspA, and their *aspA* gene deletant isogenic mutants in experiments using adherence assays to respiratory epithelium, macrophage phagocytosis and neutrophil killing assays and in vivo models of respiratory tract colonisation and infection. We demonstrated the requirement for AspA in colonization of the respiratory tract. AspA mutants were cleared from the respiratory tract and were deficient in adherence to epithelial cells, and susceptible to phagocytosis. Expression of AspA in the surrogate host *Lactococcus lactis* protected bacteria from phagocytosis. Our results suggest that AspA has an essential role in respiratory infection, and may function as a novel anti-phagocytic factor.

## Introduction

*Streptococcus pyogenes* is a Group A *Streptococcus* (GAS) that causes a wide range of diseases from pharyngitis and tonsillitis, with over 600 million cases per year [1], to more invasive diseases such as streptococcal toxic shock syndrome. *S. pyogenes* can be carried asymptomatically in the nasal cavity of humans and the nasopharynx is thought to be a primary reservoir from which *S. pyogenes* is able to infect new hosts [2]. Recently, there has been an increase in the number of cases of antibiotic resistance in *S. pyogenes*, with currently 30% of patients treated for pharyngitis failing to respond to treatment [3]. While pharyngitis itself is not life threatening it has been shown that patients left untreated can develop invasive disease and conditions such as severe acute rheumatic fever, both causing several hundred thousand deaths each year worldwide [4]. The molecular basis of *S. pyogenes* colonization of the oral cavity is unclear although factors such as M protein [5], [6], fibronectin-binding proteins [7], [8], other cell-wall anchored proteins [9] and structures such as pili [10] have all been implicated.

Some M serotypes of *S. pyogenes* e.g. M2, M4, M28 have been shown to carry a locus comprising ∼30 genes, originally designated region of difference 2 (RD2) [11]. It appears that the RD2 locus comprises a region susceptible to horizontal transfer between GAS and Group B *Streptococcus* [12]. Within the RD2 region there are four genes encoding hypothetical cell-surface anchored proteins. One of these encodes R protein, which has previously been shown to be over-represented in invasive strains of *S. pyogenes* [13], and to be involved in *S. pyogenes* adherence to epithelial cells [14]. Knock out of the R protein gene does not, however, lead to loss of invasiveness in *S. pyogenes* AL368 (M type 28) [15] suggesting that other virulence factors are involved in this process.

A second gene within the RD2 region encodes a cell surface protein (Spy_1325) that is a member of the Antigen I/II (AgI/II) family of *Streptococcus* adhesins [16]. Sequence analysis of 95 strains of *S. pyogenes* serotype M28 indicated that all strains tested carried the *spy_1325* gene [11]. The homologous genes within *S. pyogenes* MGAS6180 (M28) and MGAS10270 (M2) are 100% identical. We have recently demonstrated that Spy_1325, designated AspA [17], has many properties of the AgI/II family proteins [18]. These include the ability to bind scavenger receptor protein gp-340 [16] present at mucosal surfaces, and to mediate biofilm formation. Other proteins within the AgI/II family promote attachment and accumulation of mutans streptococci to the tooth surface [19], orchestrate interactions with other oral cavity microorganisms such as *Actinomyces naeslundii* [20] and *Porphyromonas gingivalis* [21] and bind to host receptors such as fibronectin and β-integrins mediating tissue invasion [22]. Given the evidence that AgI/II family proteins are integrally involved in host colonization by oral streptococci [9] we hypothesized that AspA might be involved in GAS colonization of mucosal surfaces.

An intranasal infection model of mice has been described for GAS serotypes M1, M14 and M49 [23], [24], [25]. Several bacterial strains caused septicaemia and mortality in different mouse strains (CD1, BALB/c, C57BL/6). However, these previous animal models were not considered to be suitable for studying longer-term GAS colonization of the oral cavity and upper respiratory tract. The carrier state, or asymptomatic infection, and persistence, are aspects of GAS biology that are poorly understood [26]. In this paper we describe a novel long-term model of upper and lower respiratory tract colonization by *S. pyogenes* MGAS10270 (M2) and MGAS6180 (M28) following intranasal infection. Both serotypes were carried asymptomatically within the nasopharynx and lungs with no development of bacteraemia following respiratory challenge. However, knockout mutants in the *aspA* gene failed to become established (being cleared by 2 days post-infection) and were rendered susceptible to macrophage phagocytosis *in vitro*. The results suggest that AspA has an important role to play in *S. pyogenes* colonization of the nasopharynx and long-term persistence of infection within the lower respiratory tract.

## Materials and Methods

### Ethics Statement

This study was performed in strict accordance with U.K. Home Office guidelines. The protocol was approved by the U.K. Home Office, and the local University of Leicester and Liverpool animal welfare and ethics committees. Every effort was made to minimize suffering and in bacterial infection experiments mice were humanely culled if they became lethargic. All animal experiments were carried out at the University of Leicester and the University of Liverpool.

### Bacterial Strains and Growth Conditions

Wild-type *S. pyogenes* MGAS6180 (M serotype 28) and MGAS10270 (M serotype 2) were routinely grown in Todd Hewitt Broth (Oxoid) containing 0.5% yeast extract and 0.5% glucose (THYG broth), and were incubated statically at 37°C under 5% CO_2_. Spectinomycin (250 µg/mL) or erythromycin (10 µg/mL) were included where appropriate. Blood agar plates consisted of Blood Agar Base (Oxoid), 5% fresh horse blood and antibiotics when necessary. Bacteria were stored at −80°C in THYG broth containing 20% glycerol. AspA^−^ mutants were constructed by allelic replacement, and complementing plasmids generated as described previously [17].

### Intranasal Infection of Mice

Female outbred MF1 mice 8–10 weeks old were lightly anaesthetized with 2.5% fluothane (AstraZeneca, Macclesfield, UK) over oxygen (1.5–2 L/min), and 50 µL PBS containing 5×10^7^ colony forming units (cfu) of *S. pyogenes* in exponential growth phase were administered to alternate nostrils. *S. pyogenes* cfu present in inocula were confirmed by viable plate counts on blood agar. At pre-determined time intervals following infection, blood was collected from groups of 10 mice per timepoint per strain under terminal anaesthesia via cardiac puncture, and mice were immediately sacrificed by cervical dislocation. Lungs, spleen, nasal associated lymphoid tissue and nasopharyngeal tissue (predominantly consisting of dorsal nasal turbinate and ventral maxilloturbinates) were dissected as described previously [27], [28] and stored in sterile, cold PBS on ice for processing. Organs were weighed and homogenized and homogenates or blood samples were serially diluted in PBS, spread onto blood agar plates with appropriate antibiotics, and cfu determined.

### Intravenous Infection of Mice

Mice were administered 0.1 mL bacteria suspension (1×10^7^ cfu) in PBS into the dorsal tail vein. At various time points post instillation, blood was taken from the dorsal tail vein to determine cfu. All animal work was carried out under appropriate UK Home Office approved personal and project licences. Mice were monitored for loss of body weight daily as well as physical symptoms at least twice daily.

### Adhesion Assays

Human lung type-II alveolar epithelial A549 cells and human pharyngeal epithelial Detroit 562 cells were provided by American Type Culture Collection (ATCC). Human lung type-II alveolar epithelial cell line A549 was maintained in DMEM (Gibco) containing 1.0 g/L glucose, 10% FCS, penicillin and streptomycin. Cells were detached using trypsin and seeded onto 24 well, tissue culture treated plates at 10^5^ cells per well. Cells were incubated for 2 d to form 70% confluent monolayers. Detroit 562 cells (human pharyngeal epithelial cell line) were maintained in MEM (Gibco) with 10% FCS, penicillin and streptomycin, and monolayers were formed as above. Sixteen hours before commencing co-culture with bacteria, tissue culture media were changed to DMEM or MEM respectively containing 10% FCS but without antibiotics. Bacteria were harvested from 18-h cultures by centrifugation (8000×g, 10 min), washed twice with sterile, endotoxin-tested DPBS (plus Mg^2+^ and Ca^2+^) and suspended at 10^6^ cfu/mL in DMEM or MEM with FCS. Cultured cells were washed 3 times with sterile PBS and 10^6^ cfu *S. pyogenes* were added to each well. Control wells contained bacteria or cells only. After 2 h incubation at 37°C in 5% CO_2_, wells were washed 5 times with PBS to remove unattached bacteria. Cells were then detached following incubation with trypsin (10 min), lysed with 0.025% Triton X 100, and samples were plated onto blood agar for determining cfu.

### Killing Assays

The human neutrophil (HL60) and mouse macrophage (J774.2) cell lines, provided by American Type Culture Collection (ATCC), were utilized in keeping with previously published standardized reference Opsono-Phagocytosis Killing Assays (OPKA) [29], [30]. In brief, differentiated HL60 cell suspensions in HBSS (10 µl suspension) were stained using Trypan blue (1∶1) for viability and counted utilizing a haemocytometer. *S. pyogenes* cells (10^7^ cells/mL in HBSS-20% serum) were opsonized for 30 min at 37°C with shaking, collected by centrifugation and suspended in HBSS (10^4 ^cfu/mL). Bacteria were incubated with 10^5^ neutrophils for 1 h and cfu were determined by blood agar plate count. The J774.2 murine macrophage cell line was maintained as previously described [31]. Briefly, 5×10^4^ macrophages were incubated with 5×10^3^ opsonized *S. pyogenes* for one hour at 37°C (5% CO_2_) and cfu were established as above. Wells containing non-opsonized streptococci and heat-inactivated complement were used as controls. Percentage killing was calculated from cfu remaining compared to control samples without phagocytic cells.

### Statistics

All statistics were carried out using Graph Pad Prism 5. Data are expressed as mean plus standard error of the mean (SEM). Statistical analysis was carried out using 2-tailed, unpaired Student t-test, with significance at *P*<0.05.

## Results

### Colonization of the Respiratory Tract by GAS

**Figure 1:**
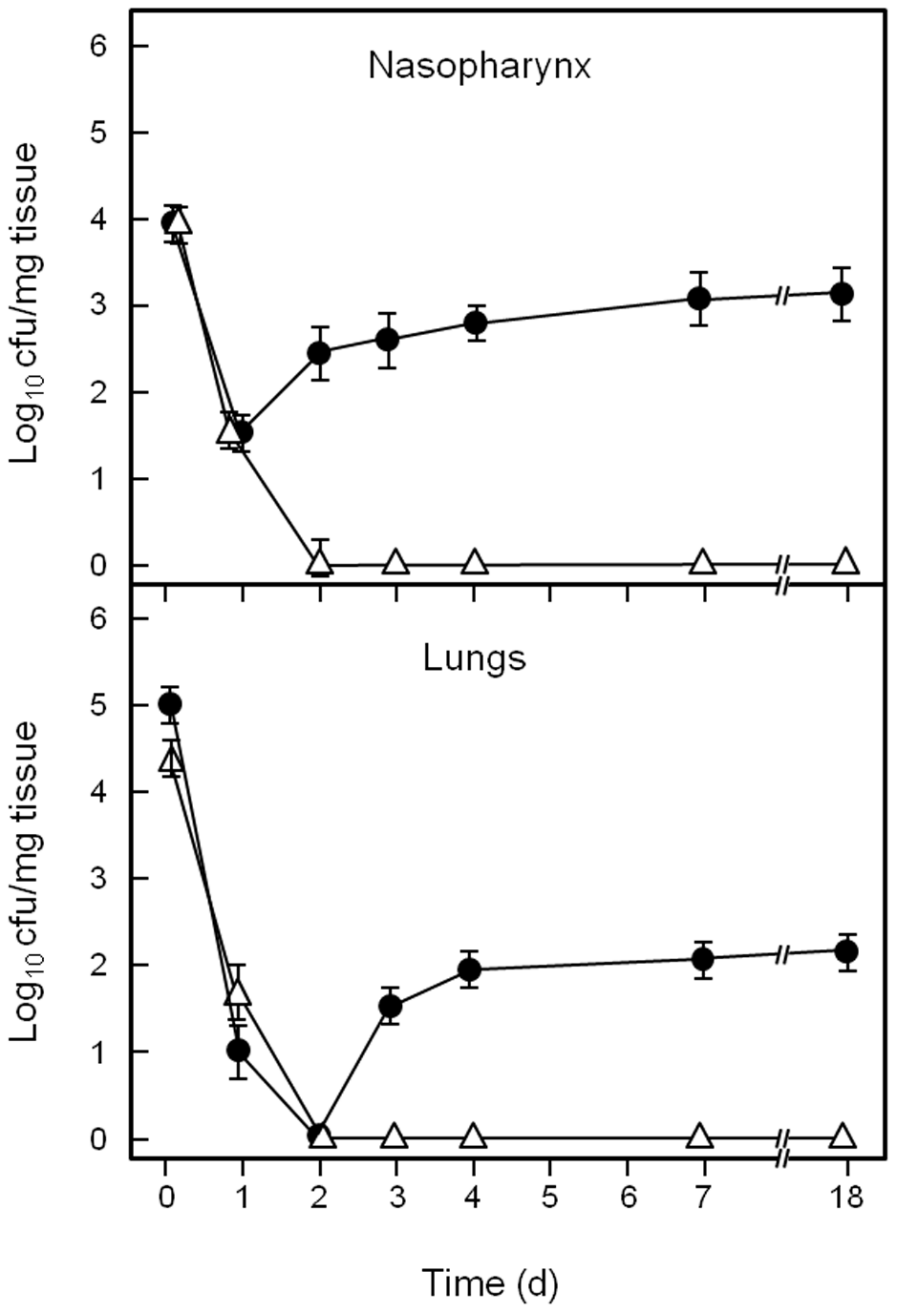
Bacterial numbers (cfu) within nasopharynx or lungs at various times following intranasal challenge of mice (1×10^8^ cells) with *S. pyogenes* MGAS10270 (•) or MGAS10270 *aspA* (Δ) (UB2115). Data are means ± SEM of 3 experiments.

**Figure 2:**
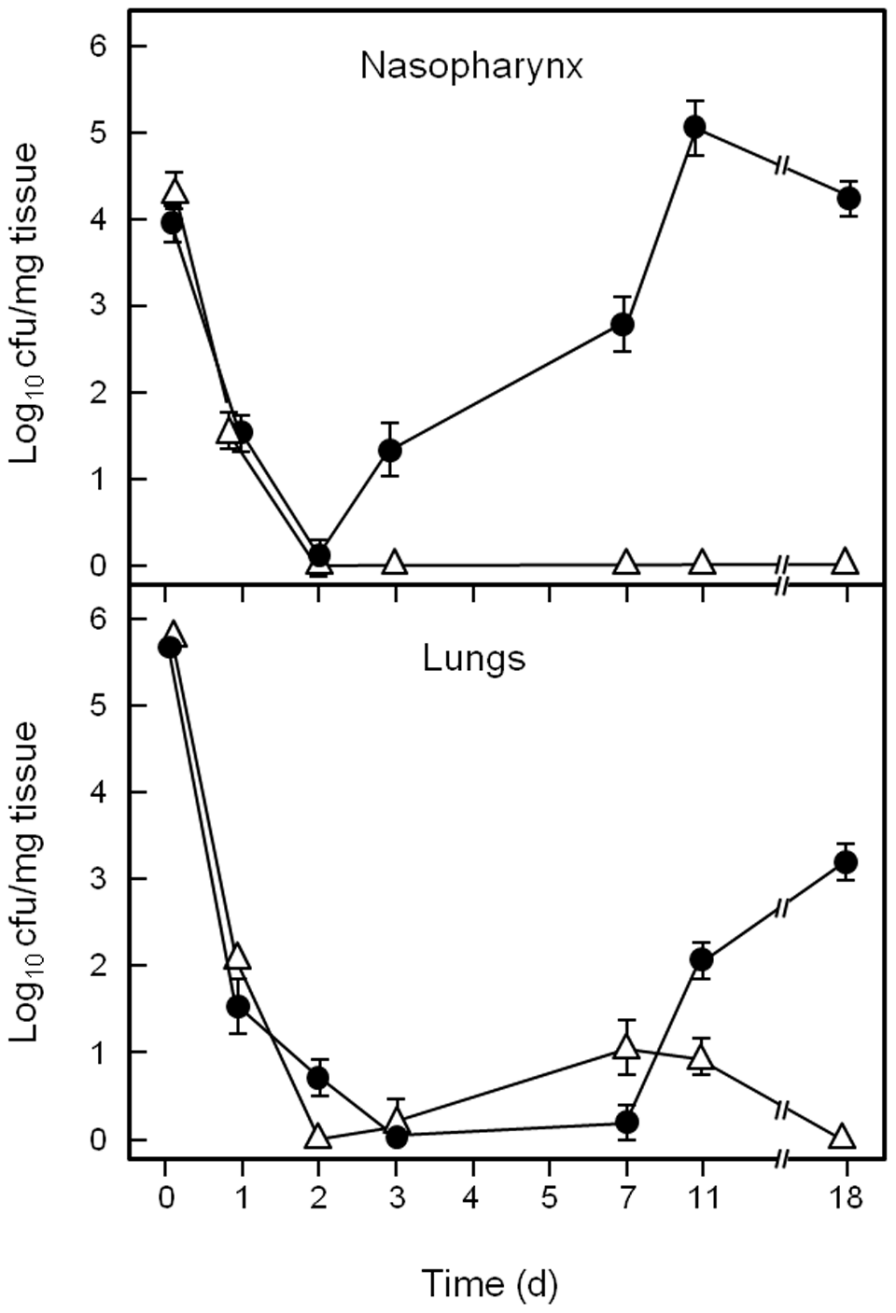
Bacterial numbers (cfu) within nasopharynx or lungs at various times following intranasal challenge of mice (1×10^8^ cells) with *S. pyogenes* MGAS6180 (•) or MGAS6180 *aspA* (Δ) (UB2086). Data are means ± SEM of 3 experiments.

With knowledge gained from design of a long-term colonization model for *Streptococcus pneumoniae* [27], [32], we developed a respiratory tract colonization model for *S. pyogenes*. Mice were challenged intra-nasally with 5×10^7^ cells of *S. pyogenes* MGAS10270 or MGAS6180, and were sacrificed at various time points post-infection. Colony forming units (cfu) of *S. pyogenes* within the nasopharynx and lungs were then determined. Bacterial cfu were also determined in blood, nasal associated lymphoid tissue (NALT) and spleen, but all of these tissues were negative for bacteria (data not shown). For both MGAS10270 and MGAS6180 strains, cfu present in the nasopharynx and lungs decreased significantly over the first 24–48 h post-infection compared to time zero (*P*<0.01) (Fig. 1, Fig. 2). However, at 48 h, levels of MGAS10270 in the nasopharynx began to increase, followed by an increase in cfu in the lungs by 72 h (p<0.05 compared to previous time points) (Fig. 1). Strain MGAS10270 cfu continued to increase in the nasopharynx up to day 7 and then remained at a level of approximately 10^3^ cfu/mg tissue until day 18 (Fig. 1). Levels of MGAS10270 within the lungs were consistently lower than those in the nasopharynx, however by day 18 all animals tested still had 10^2^ cfu/mg within their lungs.

Mice infected with MGAS6180 cleared the majority of bacteria from the nasopharynx and lungs within the first 48 h (*P<*0.01 compared to time zero) (Fig. 2). In a similar pattern to MGAS 10270, cfu in the nasopharynx began to increase by 72 h post-infection, followed by an increase by day 11 in MGAS6180 cfu in the lungs *(P<*0.05 compared to previous time points). Bacteria present within the nasopharynx reached a peak of approximately10^5^ cfu/mg tissue at day 11 and colonization was retained in the nasopharynx and lungs to at least day 18 (Fig. 2). Blood samples taken from mice infected with either strain of *S. pyogenes* were negative for cfu at all time points tested.

During bacterial colonization of the lower respiratory tract with both GAS strains, we observed significant increases in lung macrophage levels in infected mice (data not shown). The increases in macrophage numbers (over 10-fold than time zero) correlated with reductions in cfu during the first 24–48 h for both strains. Mice colonized with strains MGAS10270 and MGAS6180 exhibited small increases in the numbers of neutrophils within their lungs at 24 h post-infection compared to time zero, but by 72 h post-infection these numbers had returned to pre-infection levels (data not shown). Overall, there were no significant differences in lung neutrophil infiltration between the two wild type strains. There were also no significant increases in lymphocyte levels.

### Respiratory Tract Infection with *aspA* Mutants

In contrast to the wild type strains, Δ*aspA* mutants were unable to colonize the nasopharynx or lungs of infected mice (Fig. 1, Fig. 2). The Δ*aspA* mutant (UB2115), derived from MGAS10270, was cleared from both the nasopharynx and lungs by 48 h post-infection (Fig. 1). The Δ*aspA* mutant (UB2086) derived from MGAS6180 was also cleared by 48 h post-infection. There was a transient appearance of cfu in lungs from day 3–7, but thereafter the bacteria were cleared (Fig. 2). No *aspA* mutant cfu were found in the blood, spleen or NALT (data not shown).

The abilities of complemented Δ*aspA* mutants, carrying plasmid pKS80 expressing *aspA* [17], to colonize the murine upper respiratory tract were also tested. Although these strains over-expressed AspA [17], they were unable to become established longer term (data not shown). The few cfu obtained from mice at day 7 were erythromycin-sensitive, indicating loss of the complementing plasmids, confirmed by PCR analysis with *ermAM* primers. This implied that there was insufficient selection pressure to retain the complementing plasmids under the conditions tested.

### Sepsis Model of *S. pyogenes* Infection

Since it was not possible to detect bacteria in the blood following respiratory infection with either wild type or Δ*aspA* mutant strains, this raised the possibility that they were unable to survive in blood, or that they did not translocate from lungs into blood in sufficient enough numbers. To test this, mice were intravenously administered with 10^7^ cfu wild type or Δ*aspA* mutant strains. Bacteria were capable of surviving in blood over a 7-day study period with no significant differences between wild type or Δ*aspA* mutant strains (data not shown). These results suggested that all strains were able to survive in blood when directly administered and were not cleared by 7 days post-infection. Therefore, GAS strains are either retained within the respiratory tract following intranasal challenge and do not translocate into blood or if they do, they do so in very small numbers, which cannot be detected.

### Adhesion to Respiratory Epithelial Cells

**Figure 3:**
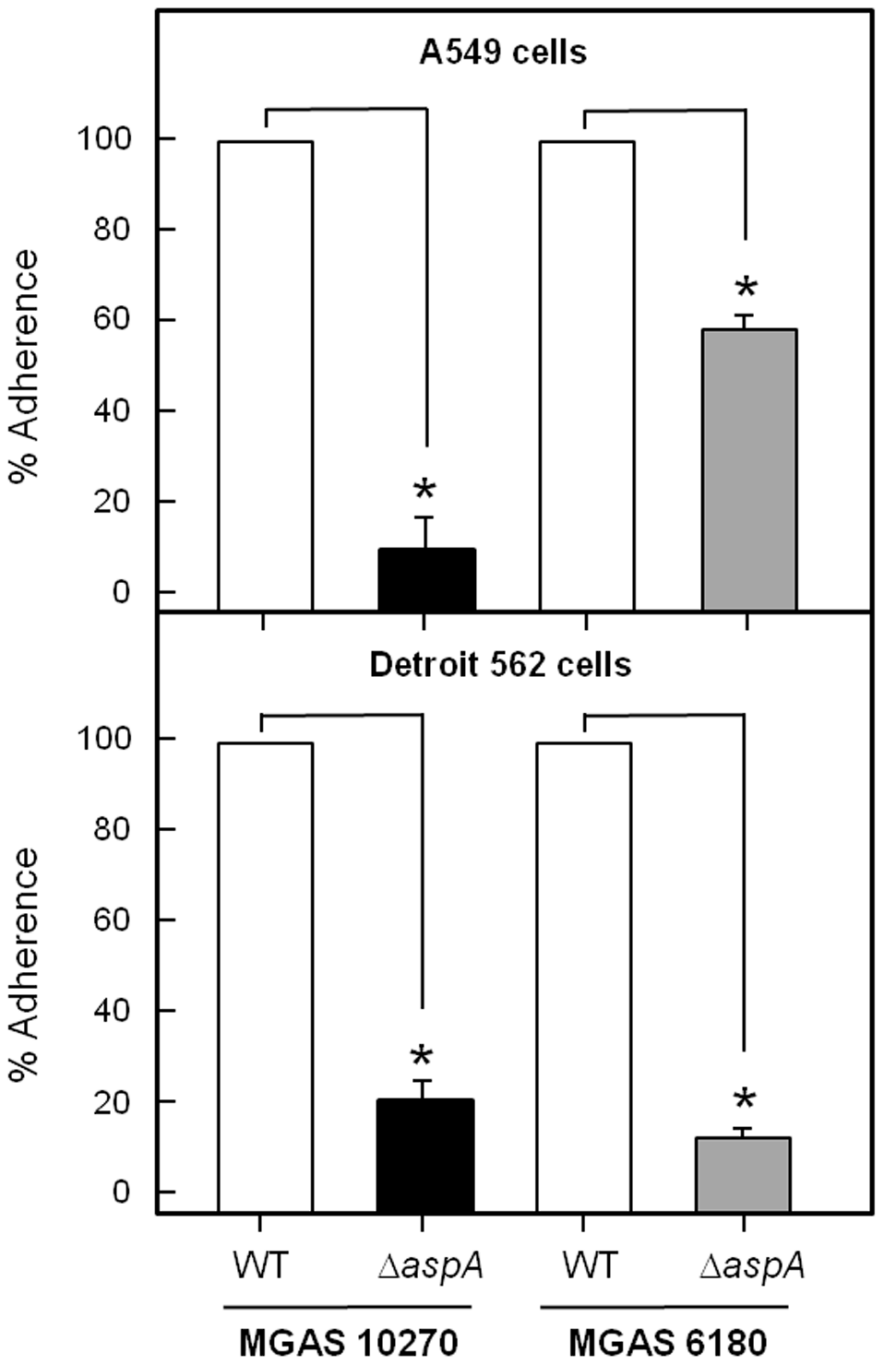
Adherence levels of MGAS10270 or MGAS6180 and corresponding Δ*aspA* mutants to A549 lung epithelial cells (pneumocytes) or Detroit 562 pharyngeal epithelial cells. Adherence is expressed as percentage of input cells (10^6^ cfu) attached. Data are means ± SEM of 3 experiments repeated in triplicate. * = *P*<0.001 using 2 tail student T-test.

The AgI/II family polypeptides have a wide range of adhesion properties, including the ability to mediate streptococcal adherence to epithelial cells [18], [22]. To test therefore the hypothesis that AspA might be an epithelial cell adhesin, the abilities of the wild-type and isogenic Δ*aspA* mutant strains to adhere to two different epithelial cell lines was investigated. Strain UB2115 showed 80–90% reduced adherence levels to A549 lung pneumocytes, and to Detroit 562 laryngeal epithelial cells, compared to MGAS10270 parent (Fig. 3). Likewise, UB2086 exhibited >90% reduced levels of adherence to Detroit cells, compared to MGAS6180, but only 40% reduced levels of adherence to A549 cells (Fig. 3). Overall, the Δ*aspA* mutant strains were significantly (*P<*0.001) less able to adhere to nasopharyngeal or lung epithelial cells compared to their parent wild-type strains. The overall adherence levels of MGAS10270 to the cell lines were five times greater than those of MGAS6180.

### Susceptibility of GAS to Phagocytic Killing

**Figure 4:**
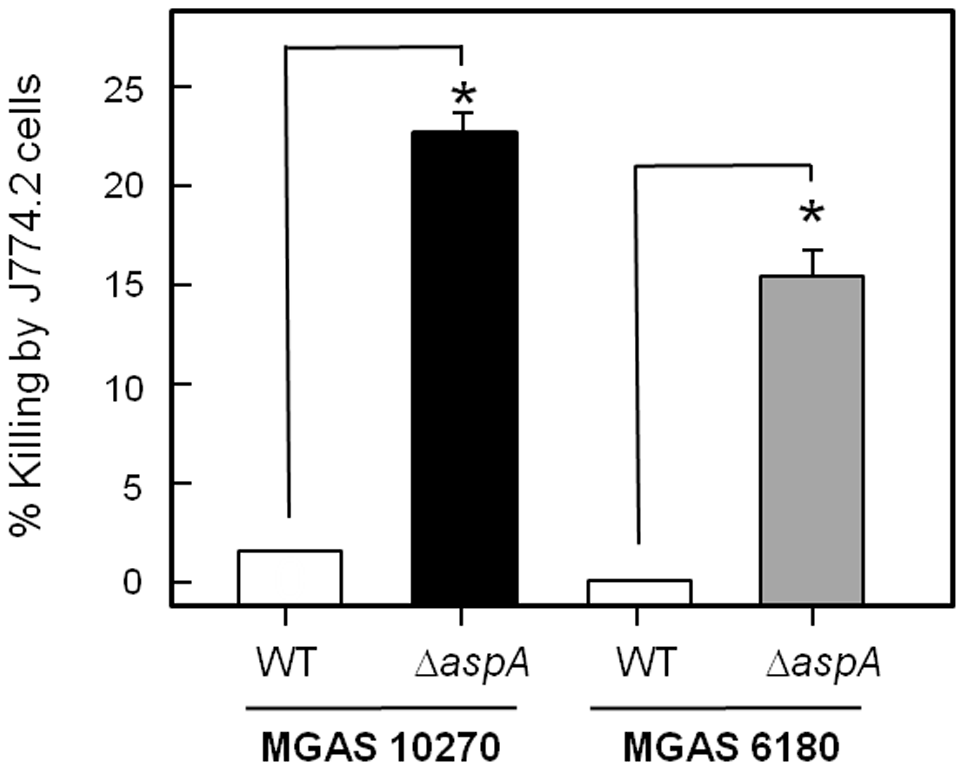
Numbers of bacteria (cfu) killed by macrophages (J774.2 cell line) following 1 h co-incubation with *S. pyogenes* MGAS6180 or MGAS10270 wild type and *aspA* mutants (input 5×10^3^ cfu, 5×10^4^ macrophages). Percentage killing was calculated from cfu remaining compared with control samples without macrophages. Data are means ± SEM of 3 experiments repeated in triplicate. * = *P*<0.001 using 2 tail student T-test.

GAS strains are known to produce a variety of anti-phagocytic factors, such as M protein and capsular polysaccharide [33]. To determine if AspA played a role in protecting cells from phagocytic killing, opsonophagocytosis killing assays with mouse macrophage J774.2 cells were performed comparing wild-type and Δ*aspA* mutant strains. Cells of MGAS10270 and MGAS6180 were killed by macrophages in relatively low numbers (Fig. 4) compared to controls (less than 1%). By contrast, Δ*aspA* mutant strains were killed in at least 10-fold greater numbers than the corresponding wild type strains (Fig. 4), indicating a role for AspA in protection against macrophage phagocytic killing.

**Figure 5:**
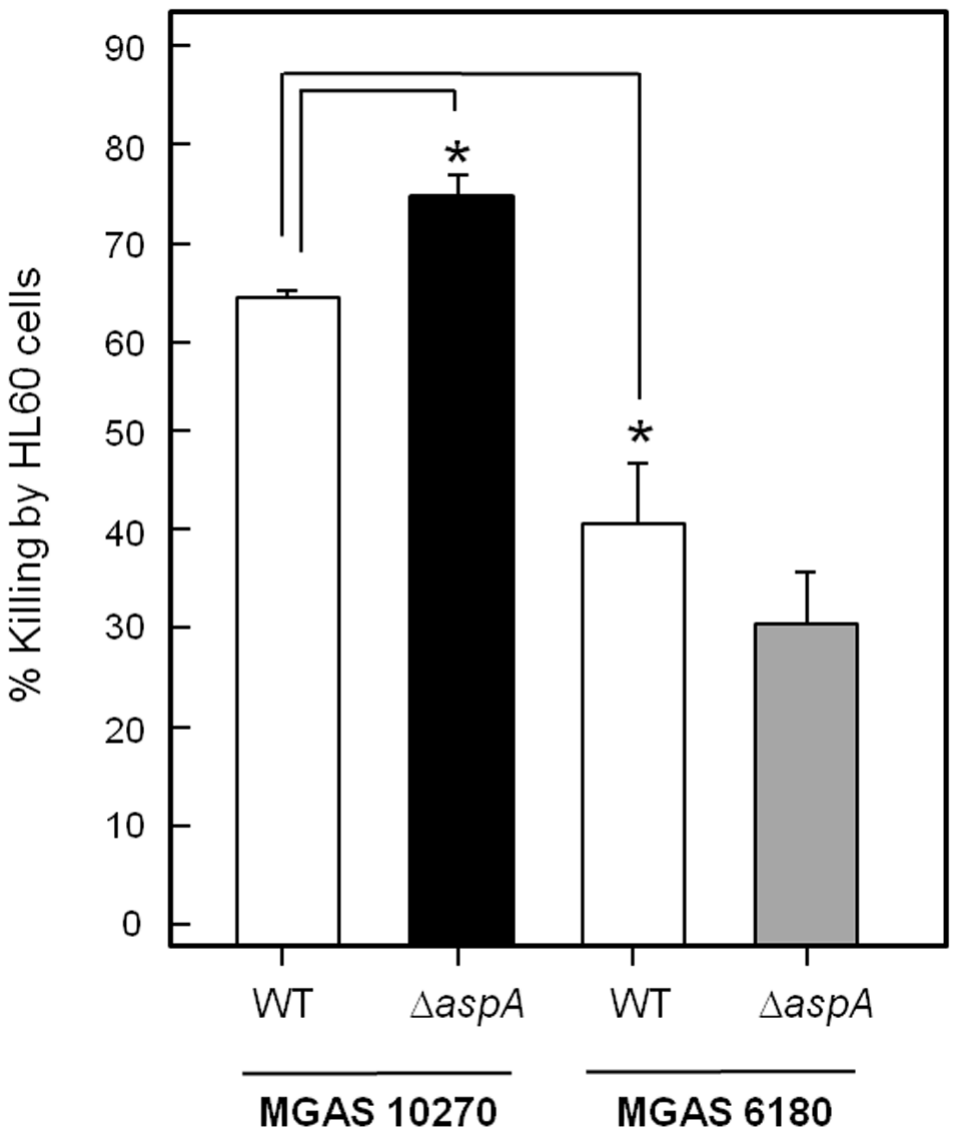
Percentage killing of *S. pyogenes* MGAS6180 or MGAS10270 wild type and *aspA* mutants by HL60 neutrophils following 1 h co-incubation as compared with controls. Percentage killing was calculated from cfu remaining compared with control samples without neutrophils (input 10^4^ cfu, 10^5^ neutrophils). Data are means ± SEM of 5 experiments repeated in triplicate. * = *P*<0.05 using 2 tail student T-test.

In neutrophil-mediated killing assays, wild-type MGAS10270 cells were more susceptible than MGAS6180 to neutrophil killing (Fig. 5). The Δ*aspA* mutant UB2115 was more sensitive to killing than corresponding wild type MGAS10270, but deletion of *aspA* from strain MGAS6180 had no significant effect on sensitivity to killing (Fig. 5).

### AspA is a New Anti-Phagocytic Factor

**Figure 6:**
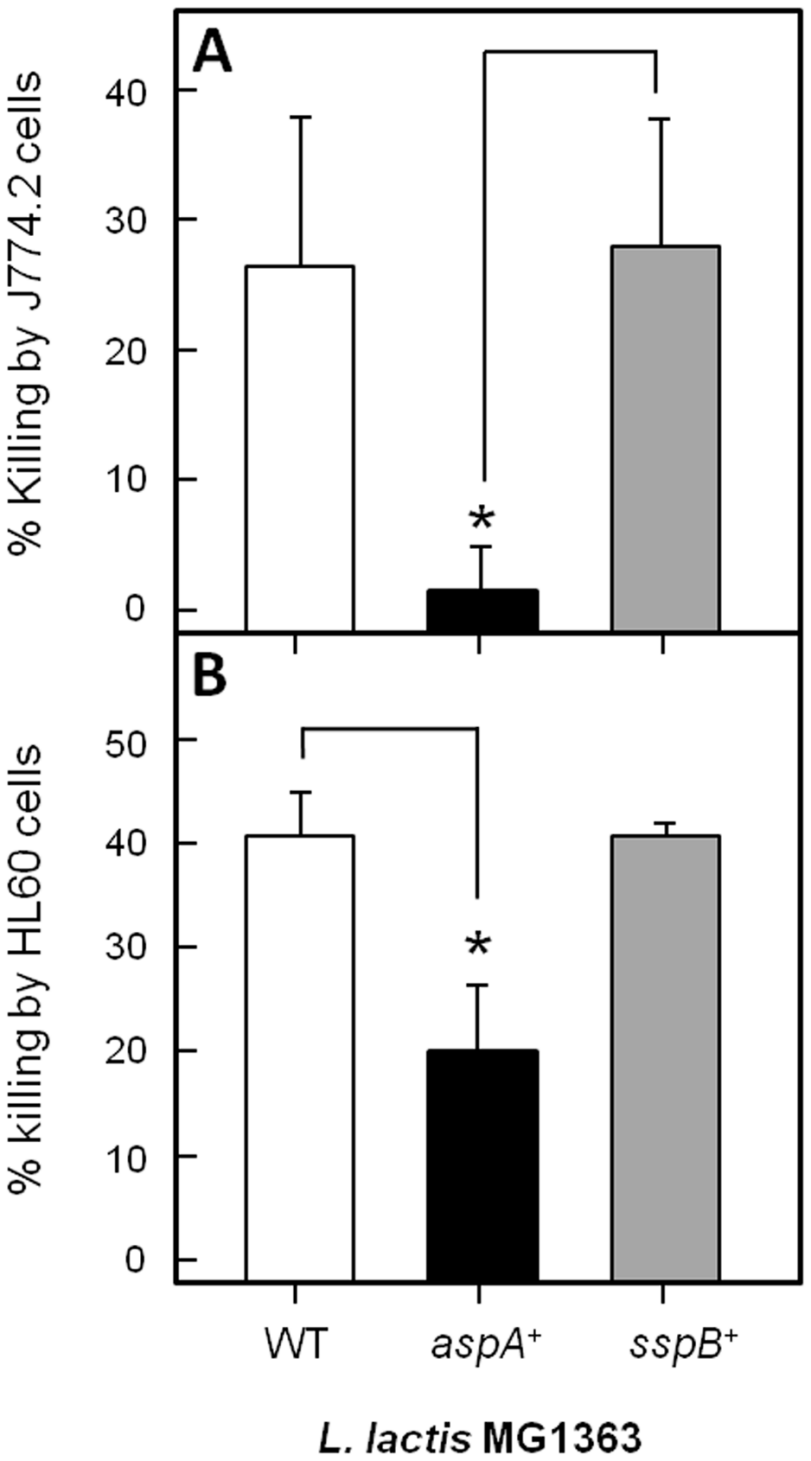
Percentage killing of *L. lactis* MG1363 wild type, and AspA or SspB expressing strains, by (A) J774.2 macrophages or (B) HL60 neutrophils following 1 h co-incubation compared with controls. Percentage killing was calculated from cfu remaining compared with control samples without macrophages or neutrophils. Data are means ± SEM of 3 experiments repeated in triplicate. * = P<0.05 using 2 tail student T-test.

To determine if the AspA protein alone had anti-phagocytic killing function, the *aspA* gene was cloned in plasmid pKS80 and expressed on the surface of *L. lactis* MG1363 [17]. Wild-type *L. lactis* cells were rapidly phagocytosed and killed by J774.2 macrophages whereas the AspA expressing cells were protected against internalization (Fig. 6A). *L. lactis* cells expressing SspB polypeptide, AgI/II family protein from *S. gordonii*, were killed at levels similar to those of the *L. lactis* vector control strain (Fig. 6A). This identifies AspA as a novel AgI/II family polypeptide with anti-phagocytic killing properties. In neutrophil-mediated killing assays, the AspA-expressing strain of *L. lactis* was less susceptible to neutrophil killing than either the vector-only or SspB-expressing strains (Fig. 6B). This suggests that AspA expression may also contribute to reducing sensitivity to neutrophil-mediated killing.

## Discussion

In recent years there has been an increase in *S. pyogenes* resistance to antibiotics in treatment of upper respiratory tract infections. This allows *S. pyogenes* the opportunity to form a stable reservoir in the nasopharynx, from which it can cause invasive acute infections [3]. Currently there is no long-term animal model of *S. pyogenes* respiratory tract colonization to study these interactions in detail. In this paper we describe a new long-term asymptomatic respiratory tract colonization model for *S. pyogenes* using serotype M2 and M28 strains. Bacteria colonized the nasopharynx and lungs of mice for 18 days without bacteraemia and with no signs of illness, with all mice surviving their infections. Interestingly, of the two wild-type strains tested, MGAS6180 had lower numbers of bacteria colonizing the nasopharynx and lungs at the 48–72 h period than MGAS10270. Thereafter however, cfu increased for both strains and remained stable. Since MGAS10270 was isolated from a pharyngitis case, it is possible that it is better adapted for respiratory tract colonization than invasive MGAS6180. However, previous studies have also shown that mutations in the two-component regulatory system CovR/S can generate GAS strains with increased invasive potential [34]. Modulation in expression of a number of virulence factors, including SpeB, hyaluronic acid capsule and streptolysin O, confers an increased capacity to evade immune defences and impaired attachment to host tissues [35], [36], [37]. Of the strains we used, MGAS10270 possesses wild-type CovR/S, whilst MGAS6180 carries a single amino acid substitution in CovS (E226G). Future studies will determine if the CovS mutation in MGAS6180 directly affects the relative expression levels of AspA in vivo between these two strains. Ultimately, however, both strains successfully colonized the upper and lower respiratory tracts.

Deletion of *aspA* caused the bacteria to be cleared from the nasopharynx and lungs of mice. The MGAS10270 Δ*aspA* mutant was essentially cleared 48 h post-infection, while MGAS6180 Δ*aspA* mutant was not completely cleared from the lungs until sometime after day 11 post-infection. The delay in reappearance of the MGAS6180 wild-type in the lungs might be because, being an invasive strain isolated from a case of sepsis, it is less well adapted to colonizing respiratory host tissue. Ultimately, both Δ*aspA* mutants were cleared providing strong evidence that this protein plays an important role in GAS colonization and survival *in vivo*.

*In vitro* analyses showed that Δ*aspA* mutants were significantly less able to adhere to respiratory tract cells. This may have impacted, at least in part, on the abilities of the mutants to become established *in vivo*. Furthermore, Δ*aspA* mutants were killed by mouse macrophages at significantly greater levels than their corresponding wild-type parents, providing a possible explanation as to why the mutants were cleared significantly quicker than the wild type strains *in vivo*. This would suggest that AspA has a role in protection of bacteria against phagocytic killing by macrophages. Our previous studies have shown that AspA is necessary for biofilm formation by MGAS6180 [17] and so one possible explanation is that because AspA sufficient bacteria form biofilms they are better protected from phagocytosis.

By expressing AspA on the surface of *L. lactis* cells we discovered that the protein itself had anti-phagocytic properties. AspA protein was protective against phagocytic killing of *L. lactis* cells, while expression of the related AgI/II protein SspB from *S. gordonii* was not protective. The expression strains have been previously described and characterized [17] and shown to have identical adherence levels to salivary agglutinin gp-340, a ligand recognized by all known AgI/II proteins to date [18]. The major differences in primary structure between the AspA and SspB proteins reside within the centrally-located V (variable) region. Since the sequences and predicted structures of other regions within AspA and SspB are similar [18], it seems likely that the V region may contribute, at least in part, to the anti-phagocytic properties of AspA.

Anti-phagocytic functions have been attributed to a number of surface or secreted proteins in GAS, including M protein [38], [39], [40], [41], protein H [42], C5a peptidase ScpA [43], IgG endoglycosidase EndoS [44], [45], cysteine protease SpeB [46], [47], Scl1 [48], GAP3DH [49], and Mac [50], [51]. Some of these function as anti-phagocytic factors by binding plasma components, thus camouflaging or blocking the bacterial cells from phagocytic killing [30], [38]. Others function by degrading immunoglobulins [51] or complement factors [43] required for opsonisation [52]. The molecular mechanism by which AspA protects against phagocytic killing is currently under investigation. We have, to date, been unable to assign any proteolytic activity or fibrinogen-binding properties to AspA.

Unexpectedly, AspA also appeared to have a direct effect on protecting *L. lactis* cells against neutrophil-mediated killing, while *S. pyogenes* Δ*aspA* mutants were unaffected in their susceptibility to neutrophil-mediated killing. This implies that other factors on the GAS cell surface were sufficient to provide protection under the conditions of our assays. During bacterial colonization of the lower respiratory tract with both MGAS strains, we observed significant increases in lung macrophage levels (although not neutrophils) that correlated with reductions in cfu during the first 24–48 h for both strains. This suggests a process whereby there was initially successful phagocytosis of colonizing bacteria by macrophages, but not complete clearance. AspA could be an essential survival factor if expression was up-regulated in bacteria colonizing mucosal surfaces. However, relative expression levels of AspA and other surface proteins *in vivo* are not known at this time.

**Figure 7:**
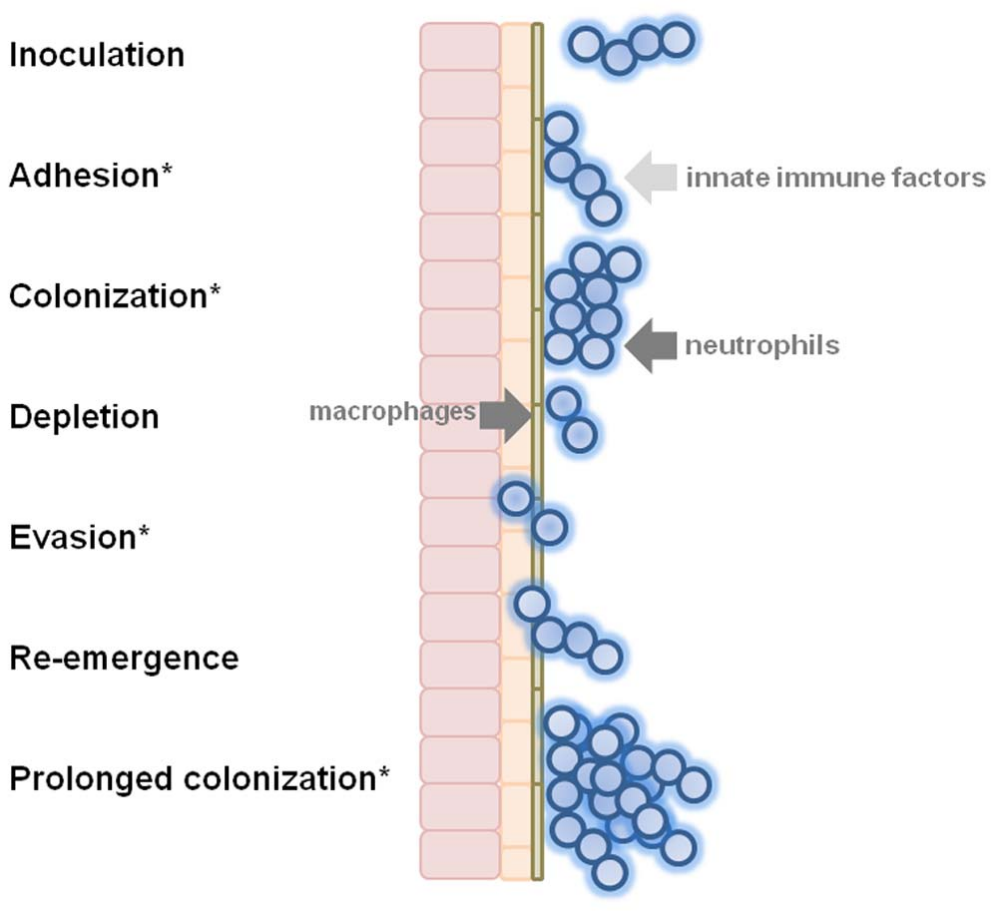
Model for the establishment of longer-term respiratory tract colonization by GAS. Following adhesion of bacterial cells to epithelium, and initial transient colonization, there is depletion of bacterial numbers due to host immune responses. These include innate factors, such as anti-microbial peptides and agglutinins, neutrophils and macrophages. A small number of bacterial cells successfully evade these responses, perhaps associated with up-regulation of AspA or transient internalization by epithelial cells. Expression of AspA brings into play the anti-phagocytic properties and biofilm-enhancing activities of AspA, leading to prolonged colonization of the mucosa. Asterisks denote temporal role for AspA.

A summary of the proposed functions of AspA is depicted in Fig. 7. Overall, by utilizing a new model of long-term pulmonary colonization, we have shown that *S. pyogenes* can colonize both the nasopharynx and lungs of mice and that AspA has an essential role in successful colonization. Together with *in vitro* and *ex-vivo* evidence we show that AspA increases adherence to respiratory epithelial cells and decreases levels of macrophage phagocytosis and neutrophil killing. These attributes identify AspA as a novel colonization and anti-phagocytic factor in GAS. In these respects, AspA protein may provide *S. pyogenes* with the ability to more successfully compete with other colonizing bacteria at the mucosal surface, as well as protection against innate immune defenses. Development of strategies aimed at blocking AspA functions might therefore be useful in controlling or preventing colonization of the upper respiratory tract by GAS.
