# Correction: The AgI/II Family Adhesin AspA Is Required for Respiratory Infection by *Streptococcus pyogenes*

**DOI:** 10.1371/annotation/8b573590-d849-46b0-8cdf-277d125b4478

**Published:** 2013-09-17

**Authors:** Linda Franklin, Angela H. Nobbs, Laura Bricio-Moreno, Christopher J. Wright, Sarah E. Maddocks, Jaspreet Singh Sahota, Joe Ralph, Matthew O’Connor, Howard F. Jenkinson, Aras Kadioglu

In the Author Contributions statement, the second author (AHN) was incorrectly omitted from the list of authors who wrote the paper. 

